# The Clinics of John B. Murphy, M. D.

**Published:** 1915-04

**Authors:** 


**The Clinics of John B. Murphy, M. D.,** at Mercy Hospital, Chicago. Volume IV, Number 1. (February, 1915). Octavo of 185 pages, 41 illustrations. Philadelphia and London: W. B. Saunders Company, 1915. Published Bi-Monthly. Price per year. Paper, $8.00. Cloth, $12.00

The February number of the clinics contains an address by Dr. Harvey R. Gaylord of Buffalo, N. Y., on the the Relation of cancer research to the clinical aspects of cancer. The address was delivered in Chicago evidently at Mercy Hospital. Dr. Murphy in introduction calls him one of the master controlling minds in the research work in the problem of cancer, and perhaps the guiding spirit in this work in America. The address covers 12 pages. This number includes talks and clinics on Intestinal Fistula, Carbuncles, Contracting cicatrix of index-finger, aneurism of trachial artery, etc., including much bone and joint work.—C. W. H.

